# The impact of COVID-19 pandemic on colorectal cancer patients: a single-center retrospective study

**DOI:** 10.1186/s12876-021-01768-8

**Published:** 2021-04-20

**Authors:** Yun Xu, Zong-Hao Huang, Charlie Zhi-Lin Zheng, Cong Li, Yu-Qin Zhang, Tian-An Guo, Fang-Qi Liu, Ye Xu

**Affiliations:** 1 Department of Colorectal Surgery, Fudan University Shanghai Cancer Center, Dong’an Road, 270, Shanghai, 200032 China; 2 Department of Oncology, Shanghai Medical College, Fudan University, Shanghai, 200032 China; 3 Hospital Information Centre, Fudan University Shanghai Cancer Center, Dong’an Road, 270, Shanghai, 200032 China; 4 Mechanical and Aerospace Engineering, University of California, 7400 Boelter Hall, Los Angeles, CA 90095 USA

**Keywords:** Public health emergency, Colorectal surgery, Endoscopy, Chemotherapy, Clinical management

## Abstract

**Background:**

Since December 2019, China has experienced a public health emergency from the coronavirus disease, which has become a pandemic and is impacting the care of cancer patients worldwide. This study evaluated the impact of the pandemic on colorectal cancer (CRC) patients at our center and aimed to share the lessons we learned with clinics currently experiencing this impact.

**Methods:**

We retrospectively collected data on CRC patients admitted between January 1, 2020 and May 3, 2020; the control group comprised patients admitted between January 1, 2019 and May 3, 2019.

**Results:**

During the pandemic, outpatient volumes decreased significantly, especially those of nonlocal and elderly patients, whereas the number of patients who received chemotherapy and surgery remained the same. During the pandemic, 710 CRC patients underwent curative resection. The proportion of patients who received laparoscopic surgeries was 49.4%, significantly higher than the 39.5% during the same period in 2019. The proportion of major complication during the pandemic was not significantly different from that of the control group. The mean hospital stay was significantly longer than that of the control group.

**Conclusions:**

CRC patients confirmed to be infection-free can receive routine treatment. Using online medical counseling and appropriate identification, treatment and follow-up can be effectively maintained. Adjuvant and palliative chemotherapy should not be discontinued. Endoscopic polypectomy, elective, palliative, and multidisciplinary surgeries can be postponed, while curative surgery should proceed as usual. For elderly CRC patients, endoscopic surgery and neoadjuvant radiotherapy are recommended.

## Background

Since December 2019, China has experienced the public health emergency from the coronavirus disease (COVID-19) caused by the novel coronavirus [1], which is capable of human-to-human transmission through inhalation [2, 3] and potential risk of fecal–oral transmission [4–6]. The rapid spread of the virus has overwhelmed the nation’s health-care system capacity and impacted the management of cancer patients. Cancer patients have been reported to have higher susceptibility to viral infection, adverse events, and deterioration [7, 8]. Consequently, to avoid hospital-associated viral transmission, it was recommended that treatment for most cancer patients should be delayed [9]. However, given the risks of cancer progression, delaying treatment remains controversial. A new analysis estimates that the pandemic increased cancer mortality rate by 20% [10]. Thus, within the medical community, improving the management of cancer patients during the pandemic is an urgent priority.

Colorectal cancer (CRC) is one of the most common malignant tumors worldwide [12]. As one of the largest departments of colorectal surgery in China, our center in Shanghai provides treatment for CRC patients from all over the nation, with more than 2500 cases of colorectal surgeries performed at our center annually. Meanwhile, Shanghai has the largest population flow and the largest number of top medical centers in China. Therefore, even a low number of confirmed infected cases results in the most stringent prevention and control measures across the city. To control the spread of the virus, the Shanghai Municipal People’s Government launched a first-level public health response on January 24, 2020, which introduced rigorous surveillance and safety procedures [11]. Even though our center is specialized in cancer treatment and therefore did not admit patients infected with COVID-19, the new procedures have hampered our clinical work, including both the treatment of newly diagnosed CRC patients and the continuing treatment and surveillance of postoperative patients during outpatient visits. Due to the potential risk of fecal–oral transmission, surgical treatments for CRC, including colorectal surgery and endoscopy, were severely limited during the pandemic. Moreover, administration of adjuvant chemotherapies to CRC patients was restricted as a result of their immunosuppressive effect [7]. Thus, CRC patients are experiencing an unprecedented dilemma.

In response to this dilemma, we have taken specialized measures to avoid viral infections, while ensuring the continued care for cancer patients as much as possible. These measures did not begin to loosen until April, when local infection rates substantially reduced and our clinical practice gradually returned to normal. In this work, we aimed to evaluate the impact of the pandemic on CRC patients in our center to share lessons learned for clinics currently experiencing the impact of this public health emergency.

## Methods

### Patients

We retrospectively enrolled consecutive CRC patients who received treatment at the Fudan University Shanghai Cancer Center during the pandemic (from January 1, 2020 to May 3, 2020), and a consecutive control group of patients who received treatment during the same period in 2019 (from January 1, 2019 to May 3, 2019).

### Data collection

Data on outpatient volume, drug administration, endoscopies, endoscopic treatments, and surgeries were collected from patient charts and compared between the two groups. The pandemic-related changes in CRC patient demographics, clinical care, and surgical safety were evaluated. A major complication was defined as an event requiring surgical, endoscopic, or radiologic intervention. A life-threatening complication was defined as an event requiring intermediate or intensive care and which could result in death [13].

### Measures taken in response to public health emergency

#### Outpatients

In the outpatient clinic, each patient underwent a thorough epidemiological screening to confirm that the patient has had no (1) contact with patients infected with coronavirus, (2) history of travel through areas with severe epidemics, (3) fever, cough, and other symptoms related to COVID-19 over the previous 14 days, and (4) abnormalities in routine blood and chest computed tomography (CT) examinations. When these conditions were met, the patient was confirmed as free of coronavirus infection and admitted.

We also established a telemedicine networking platform to facilitate doctor-patient communication and provide outpatient care online. We recommended nonlocal patients to take regular follow-up tests at their local hospitals. These test results were uploaded on our communication platform, and after analyzing these results, we advised patients on whether they should continue follow-ups or undergo treatment. For cases of serious adverse events, we recommended the patients to be treated at our center.

#### Nonsurgical treatments in the outpatient department

We did not restrict adjuvant chemotherapy, but intravenous chemotherapy was often replaced with oral chemotherapy in elderly patients. Chemotherapy and immunotherapy were maintained for advanced patients. Neoadjuvant radiotherapy was an option for delaying surgery for resectable rectal cancer. For patients who fulfilled the NCCN criteria, long course radiotherapy was administered. For patients who did not fulfil the NCCN criteria, short course (one week) radiotherapy was recommended. The mean waiting period was (7.1 ± 1.9) days, and all these patients proceeded to surgery afterwards.

#### Endoscopy and endoscopic treatment

For patients who underwent follow-up, nonurgent endoscopies were delayed and replaced by other imaging modalities, such as CT or magnetic resonance imaging (MRI). If a recurrence or metastasis was found, endoscopies were performed. For newly diagnosed patients, endoscopic biopsy was performed. For benign polyps, endoscopic polypectomy was postponed. For malignant pathologic results, preoperative evaluation was administered.

#### Surgical treatments

During the pandemic, elective (mainly stoma closure), palliative, and multidisciplinary surgeries were postponed. In contrast, curative resection remained routinely performed for resectable CRC patients. For elderly patients with poor general conditions, conservative treatment or palliative surgery could be performed first. Laparoscopic surgery was considered as the first option if available.

### Statistical analysis

The Statistical Package for the Social Sciences (SPSS) software (version 21.0, SPSS Inc., Chicago, IL, USA) was used for statistical analyses. Continuous variables are expressed as mean ± standard deviation and were compared using the independent-samples *t*-test. The Levene test for the homogeneity of variance were performed before *t*-test. Categorical data are expressed as numbers with percentages and were compared using the chi-squared test or the Fisher’s exact test. Two-tailed p-values less than 0.05 were considered statistically significant.

## Results

### Outpatients

**Fig. 1:**
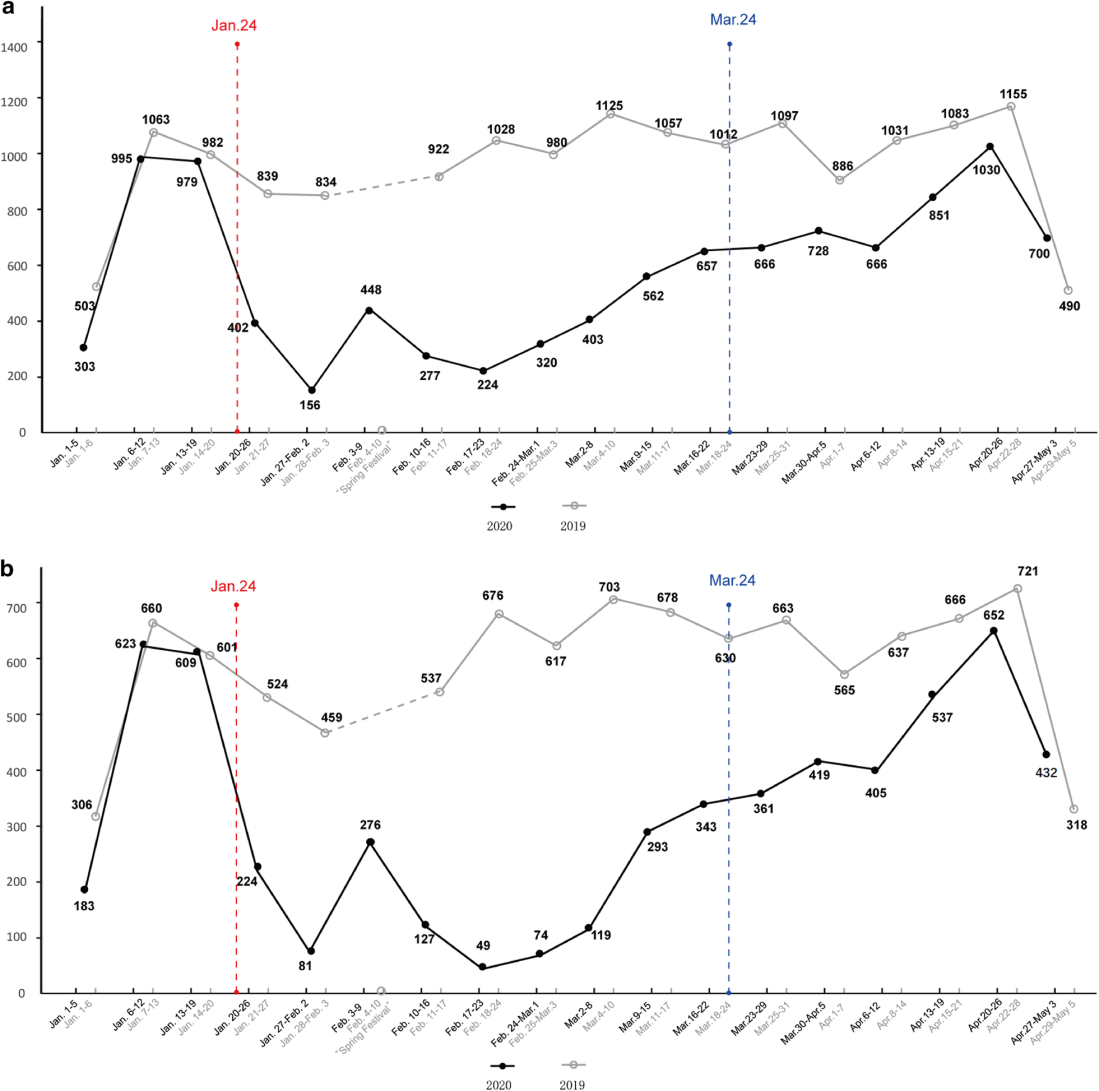
Number of outpatients per week during the pandemic. The black line illustrates outpatients from January 1 to May 3. The gray line illustrates outpatients during the same period in 2019. **a** Total outpatients; **b** nonlocal patients. The dotted line represents the holiday week of the Spring Festival. The red dotted line represents the date when the Shanghai Municipality Government announced their first-level response. The blue dotted line represents the date when the first-level response is downgraded to a second-level response

Outpatient volume during the pandemic and during the same period in 2019 are shown in Fig. 1a. During the pandemic, we received 10,367 outpatients, a significant reduction of 35.6% when compared to the same period in 2019 (16,087). The biggest reduction in outpatient volume was among nonlocal patients (5807 vs. 9961, reduced by 41.7%) (Fig. 1b). However, beginning in mid to late April, the outpatient volume gradually recovered. Through the telemedicine networking platform, a total of 3277 patients received online medical counseling. Among them, 2622 cases received follow-up after surgery and 655 cases were newly diagnosed with CRC. Among these 2622 cases, further follow-ups were recommended for 1704 cases, and treatment was recommended for 918. Of the 918 patients, 643 received adjuvant chemotherapy and 275 patients with confirmed or suggested metastasis or recurrence were recommended to be treated at our center. Among the 655 cases of newly diagnosed CRC, 459 were resectable and recommended to be treated in our hospital, while the remaining 196 unresectable cases were recommended to receive chemotherapy, immunotherapy, or radiotherapy.

### Chemotherapy in the outpatient department

**Fig. 2:**
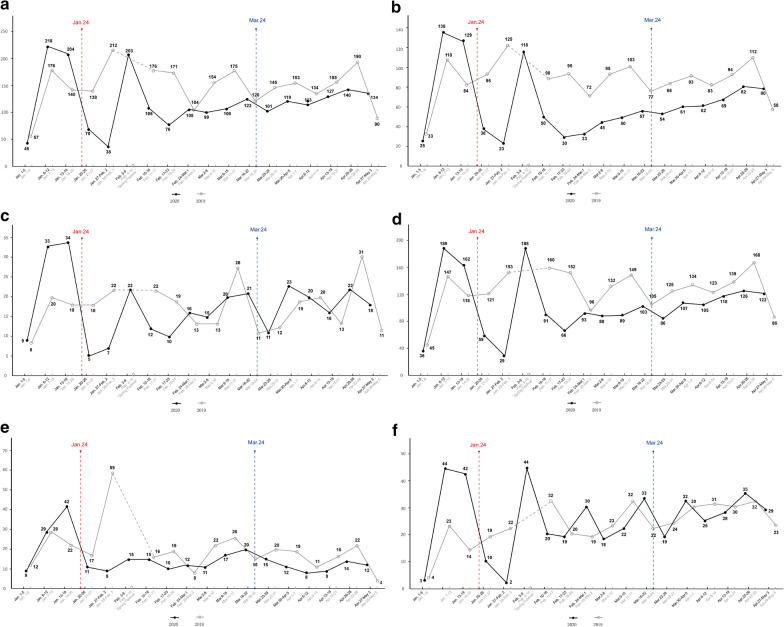
Number of patients who received chemotherapy per week during the pandemic. The black line illustrates patients who received chemotherapy from January 1 to May 3. The gray line illustrates patients who received chemotherapy during the same period in 2019. **a** Total patients; **b** nonlocal patients; **c** elderly patients; **d** patients who received intravenous chemotherapy; **e** patients who received oral chemotherapy; **f** patients who received immunotherapy

During the pandemic, 2127 CRC patients received chemotherapy in our department, including 1857 who received intravenous chemotherapy and 270 who received oral chemotherapy. This volume was 17.1% less than that of the same period in 2019 (2127 vs. 2490) (Fig. 2a), again mainly among nonlocal patients (1144 vs. 1505, reduced by 24.0%) (Fig. 2b). The volume of elderly patients (314 vs. 298) (Fig. 2c) and patients who received intravenous chemotherapy (1857 vs. 2153) (Fig. 2d), oral chemotherapy (270 vs. 337) (Fig. 2e), and immunotherapy (455 vs. 400) (Fig. 2f) largely remained at normal levels.

### Endoscopy

**Fig. 3:**
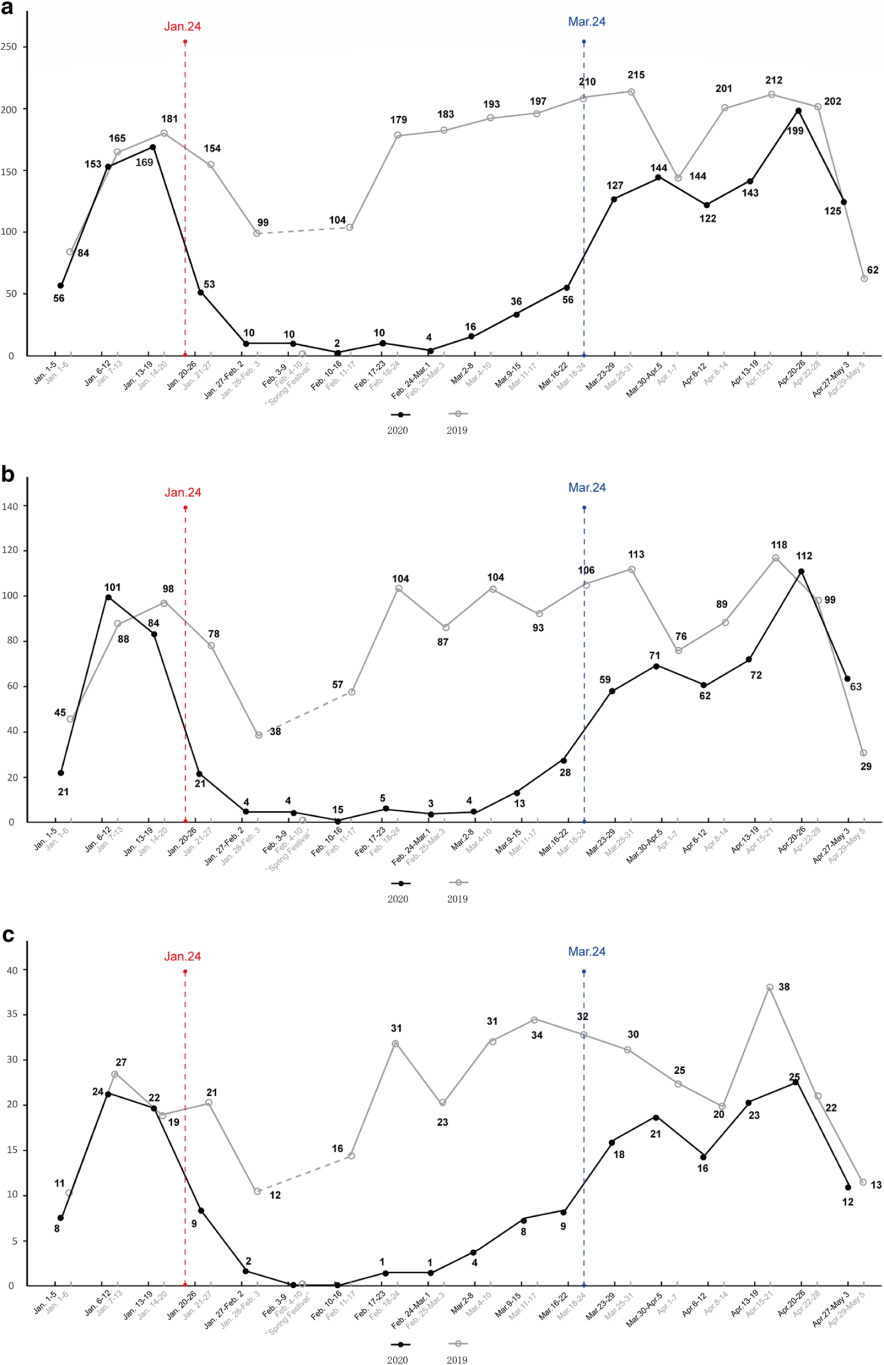
Number of patients who underwent endoscopy per week during the pandemic. The black line illustrates patients who underwent endoscopy from January 1 to May 3. The gray line illustrates patients who underwent endoscopy during the same period in 2019. **a** Total patients; **b** nonlocal patients; **c** elderly patients

During the pandemic, 1430 patients underwent endoscopy in our hospital, which was significantly lower than the volume of endoscopy tests conducted during the equivalent period in 2019 (1435 vs. 2785, reduced by 48.5%) (Fig. 3a). During the pandemic, volumes of endoscopy tests were lower among nonlocal (727 vs. 1422, reduced by 48.9%) (Fig. 3b) and elderly patients (203 vs. 405, reduced by 49.9%) (Fig. 3c). During late April, our endoscopy department resumed its normal operations, and the volume of endoscopy tests gradually recovered to normal.

### Endoscopic treatment

During the pandemic, 27 patients underwent endoscopic treatment, which was significantly lower than the volume of patients who underwent endoscopic treatment during the same period in 2019 (27 vs. 113, reduced by 76.1%) (Table 1). The number of endoscopy treatments was particularly low between February and March. Treatments for patients who were diagnosed with a colorectal polyp during this period were delayed until April.

**Table 1:** The number of patients who received endoscopic treatments per month during the pandemic and the same period in 2019

Month of year	January		February		March		April	
Year	2019	2020	2019	2020	2019	2020	2019	2020
Total volume	34	9	21	0	31	2	27	16
Migrant patients	18 (52.9)	1 (11.1)	12 (57.1)	0	15 (48.4)	0	13 (48.1)	13 (81.3)
Elderly patients	4 (11.8)	0	3 (14.3)	0	5 (16.1)	0	6 (22.2)	2 (12.5)
Begin cases	31 (91.2)	7 (77.8)	19 (90.5)	0	30 (96.8)	2 (100)	26 (96.3)	15 (93.8)
Malignant cases	3 (8.8)	2 (22.2)	2 (9.5)	0	1 (4.2)	0	1 (3.7)	1 (6.2)

### Stoma closure

During the pandemic, 91 postoperative CRC patients received stoma closure. There was a significant decrease in the number of stoma closures between January 2020 to February 2020 (Table 2). The mean hospital stay was 8.8 ± 3.1 days during the pandemic, which was significantly longer than the mean stay (6.8 ± 2.2 days) during the same period in 2019 (t = − 5.238, p < 0.001). Delayed surgeries for stoma closure were performed between late March and April.

**Table 2:** The number of patients who received stoma closure per month during the pandemic and the same period in 2019

Month of year	January		February		March		April	
Year	2019	2020	2019	2020	2019	2020	2019	2020
Total volume	33	17	17	2	23	25	24	47
Migrant patients	29 (87.9)	15 (88.2)	13 (76.5)	0	15 (65.2)	19 (76.0)	21 (87.5)	38 (80.9)
Elderly patients	6 (18.2)	2 (11.8)	5 (29.4)	0	5 (21.7)	4 (16.0)	5 (20.8)	8 (17.0)

These values are presented as number of patients followed by percentage in parentheses

### Palliative surgery

During the pandemic, 81 unresectable CRC patients received palliative surgeries (e.g., stoma formation, cytoreductive surgery, palliative resection). The number of palliative surgeries decreased in February 2020 (Table 3). During the pandemic, the mean hospital stay was 11.0 ± 4.3 days, which was significantly longer than the mean hospital stay (9.1 ± 3.1 days) during the same period in 2019 (t = − 3.087, p = 0.002).

**Table 3:** The number of patients who received palliative surgery per month during the pandemic and the same period in 2019

Month of year	January		February		March		April	
Year	2019	2020	2019	2020	2019	2020	2019	2020
Total volume	21	16	12	7	19	21	25	37
Migrant patients	18 (85.7)	15 (93.8)	10 (83.3)	1 (14.2)	16 (84.2)	7 (33.3)	22 (88.0)	27 (73.0)
Elderly patients	2 (9.5)	3 (18.8)	2 (16.7)	4 (57.1)	4 (21.1)	4 (19.0)	2 (8.0)	6 (16.2)
Laparoscopic surgery	2 (9.5)	3 (18.8)	2 (16.7)	0	1 (5.3)	4 (19.0)	7 (28.0)	6 (16.2)

These values are presented as number of patients followed by percentage in parentheses

### Multidisciplinary surgery

During the pandemic, 26 CRC patients with peripheral invasion (ovaries, uterus, fallopian tubes, urinary bladder) or liver metastasis underwent multidisciplinary surgeries. The volume of combined procedures decreased from late January to early March, mainly for elderly patients and patients with liver metastasis (Table 4). During the perioperative period, major complications occurred in three (11.5%) patients who received simultaneous resection of the CRC and liver metastasis, which was similar to complication rates in the control group (11.5% vs. 11.1%, χ^2^ = 0.245, p = 0.620). However, the mean hospital stay was 14.3 ± 4.3 days during the pandemic, which was longer than the mean hospital stay (12.3 ± 3.3 days) during the same period in 2019 (t = − 2.007, p = 0.049).

**Table 4:** The number of patients who received multidisciplinary surgery per month during the pandemic and the same period in 2019

Month of year	January		February		March		April	
Year	2019	2020	2019	2020	2019	2020	2019	2020
Total volume	14	7	8	3	5	4	9	12
Migrant patients	12 (85.7)	5 (71.4)	5 (62.5)	2 (66.7)	4 (80.0)	2 (50)	6 (66.7)	8 (66.7)
Elderly patients	3 (21.4)	0	0	0	1 (20.0)	1 (25.0)	0	2 (16.7)
Hepatic surgery	6 (42.9)	1 (14.3)	7 (87.5)	0	3 (60.0)	1 (25.0)	2 (22.2)	5 (41.7)

These values are presented as number of patients followed by percentage in parentheses

### Curative resection

**Fig. 4:**
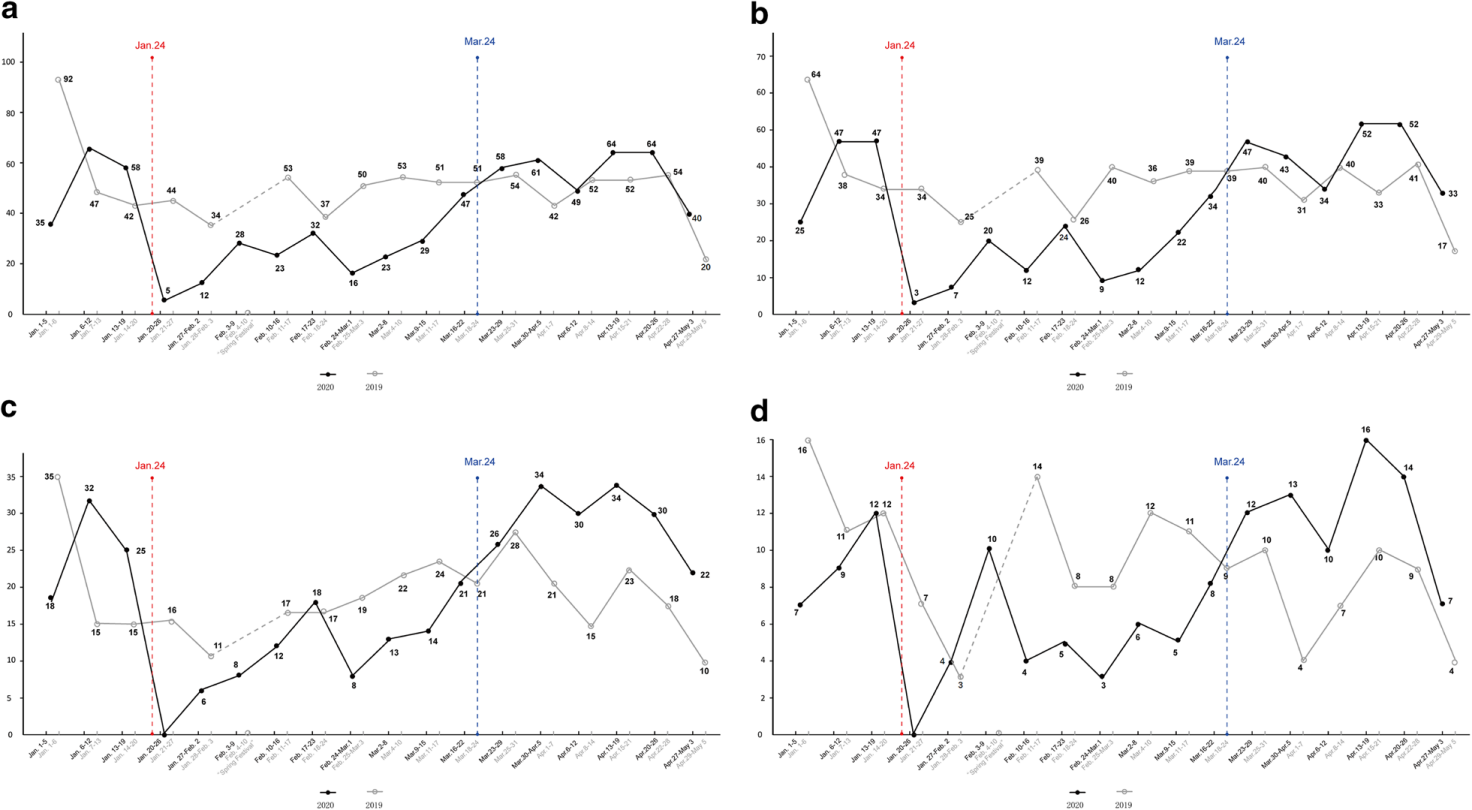
Number of patients who received curative resection per week during the pandemic. The black line illustrates patients who received curative resection from January 1 to May 3. The gray line illustrates patients who received curative resection during the same period in 2019. **a** Total patients; **b** nonlocal patients; **c** elderly patients; **d** patients who underwent laparoscopic surgery

During the pandemic, 710 CRC patients underwent curative resection. The volume of curative resection performed was reduced between late January and late March (Fig. 4a), particularly among nonlocal (Fig. 4b) and elderly patients (Fig. 4c).

Demographic and clinical parameters of patients during the pandemic and the same period in 2019 are compared and demonstrated in Table 5. During the pandemic, the proportion of performed laparoscopic surgeries was 49.4%, which was significantly higher than the proportion (39.5%) during the same period in 2019 (χ^2^ = 15.333, p < 0.001) (Fig. 4a, Table 5). In total, 17.3% (123/710) of patients underwent a stoma formation during the pandemic, which was greater than the 13.2% (109/828) who received a stoma formation in the control group (χ^2^ = 5.163, p = 0.023). Among rectal cancer patients, compared with the 17.5% (65/371) of patients who received neoadjuvant radiotherapy in the control group, a greater proportion of patients (79/333; 23.9%) received neoadjuvant radiotherapy during the pandemic (χ^2^ = 4.451, p = 0.039). The rate of major complications did not differ significantly in the perioperative period during the pandemic when compared with that of the control group (3.9% [28/710] vs. 5.1% [42/828]; χ^2^ = 1.121, p = 0.290). The mean hospital stay was 13.2 ± 4.5 days, which was significantly longer than the mean hospital stay of 11.0 ± 4.0 days during the same period in 2019 (t = − 10.298, p < 0.001). Both preoperative waiting and postoperative stays were significantly prolonged during the pandemic.

**Table 5:** Demographic and clinical parameters of patients who receive curative resection at the same period of 2019 and 2020

Parameters	2019 (N = 828)	2020 (N = 710)	χ^2^/t value	p value
Gender			0.123	0.726
Male	518 (62.6)	438 (61.7)		
Female	310 (37.4)	272 (38.3)		
Age(years)^a^			0.706	0.401
< 70	673 (81.3)	565 (79.6)		
≥ 70	155 (18.7)	145 (20.4)		
Area of origin			0.107	0.743
Local	212 (25.6)	187 (26.3)		
Migrant	616 (74.4)	523 (73.7)		
CRC location			2.336	0.311
Right colon	181 (21.9)	166 (23.4)		
Left colon	276 (33.3)	211 (29.7)		
Rectum	371 (44.8)	333 (46.9)		
Neoadjuvant chemotherapy			2.596	0.124
Received	110 (13.3)	115 (16.2)		
Neoadjuvant radiotherapy for rectal cancer			4.451	0.039
Received	65 (17.5)	79 (23.9)		
Operative methods			15.333	< 0.001
Laparoscopic	327 (39.5)	351 (49.4)		
Laparotomy	501 (60.5)	359 (50.6)		
Transfusion			0.002	0.963
Received	52 (6.3)	45 (6.3)		
Enterostomy			5.163	0.023
Received	109 (13.2)	123 (17.3)		
Major complication			1.121	0.290
Occurrence	42 (5.1)	28 (3.9)		
Preoperative waiting (d.)^a^	3.8 ± 2.8	4.8 ± 3.0	− 6.817	< 0.001
Postoperative stay (d.)^a^	7.2 ± 2.8	8.4 ± 3.1	− 7.945	< 0.001
Hospital stay (d.)^a^	11.0 ± 4.0	13.2 ± 4.5	− 10.298	< 0.001

*CRC* colorectal cancer, *d*. days

^a^These data are presented as mean ± standard deviation; other values are presented as number of patients followed by percentage in parentheses

## Discussion

The novel COVID-19 pandemic has generated substantial disruptions worldwide and impaired the ability of the hospitals to diagnose and treat cancer patients. Faced with these challenges, we enacted a series of measures, which have yielded positive results. Given cancer patients’ increased susceptibility to viral infections [7, 8], thorough epidemiological screening before outpatient admission ensured the safety of our patients. Beyond the outpatient clinic rational, selection of patients and proper allocation of resources helped us maintain most treatments involving surgery and chemotherapy for CRC patients, while focusing on routine clinical care practices and observing patient responses prevented complication rates in surgeries from increasing. On March 24, the Shanghai Municipal Government downgraded its major public health emergency first-level response to a second-level response [14]. Since then, clinical work has gradually resumed to its original state, and some of the delayed treatments were performed in April.

Since the pandemic, public transport has been restricted, and nonlocal patients have been unable to enter Shanghai for treatment. Among local patients, elderly patients are at a greater risk of infection, and consequently, many have also been unwilling to undergo treatment. These obstacles for the two groups of cancer patients to receive outpatient treatment is reflected in the significant reduction in their respective outpatient volumes after the pandemic began, as shown in this study’s results. In response, as previously discussed, we established a telemedicine networking platform to provide outpatient care and medical advice online, and only recommended online patients experiencing serious adverse events to be treated at our center. Through such online medical counseling, we effectively maintained treatment and patient follow-ups, thereby reducing mortality while ensuring the safety of patients.

Adjuvant chemotherapy and palliative chemotherapy have greatly benefitted the long-term prognosis of CRC patients. However, the immunosuppressive effects of chemotherapy have made their use during the pandemic controversial [7]. At our center, despite restricting the number of patients who received chemotherapy during the pandemic, administration of oral chemotherapy and intravenous immunotherapies were maintained at normal levels. We believe that during the epidemic, adjuvant chemotherapy should not be discontinued, given its importance for ensuring survival of CRC patients. However, intravenous chemotherapy can be discontinued and replaced with oral chemotherapy in elderly patients [15]. In addition, we believe that chemotherapy and immunotherapy must be maintained for advanced patients if the patient is confirmed to be free of infection. Otherwise, tumor-related mortality in these patients would increase.

As coronavirus is capable of fecal–oral transmission [4, 5], endoscopy may serve as a vector for viral transmission. Consequently, our center discontinued nonurgent endoscopies and endoscopic treatments from February to March, as evident in the 76.1% reduction in the number of patients that underwent endoscopic treatment after the pandemic began. This number has gradually recovered after coronavirus infection rates were significantly reduced in April. Although we believe that endoscopy can be performed selectively during the outbreak, patients must be strictly screened, and only infection-free patients should undergo endoscopy. Moreover, all medical equipment should be thoroughly disinfected to ensure patient safety. Finally, we suggest that routine endoscopy should only be performed in patients with newly diagnosed colorectal tumors or polyps who are waiting for pathological confirmation. Endoscopies for patients who are routinely monitored through follow-ups can be delayed and replaced by other imaging modalities, such as CT or MRI. If a recurrence or metastasis is found, endoscopies should be resumed. In newly diagnosed patients, endoscopic polypectomy can be postponed if polyps are small or pathologically benign.

During the pandemic, colorectal surgery experienced many problems, including a lack of available blood for transfusions. We further debated the ethical merits of treating elderly patients who may be at greater risk of viral infection if surgically treated [16–22]. Consequently, while more than 700 curative resections were performed during the pandemic, this was significant decrease from the number of surgeries performed during the same period in 2019. To ensure the safety of inpatients and medical staff, “infection-free” wards were established. In addition to a proof of admission, only patients and their accompanying guests who presented a proof of stay in Shanghai for 14 days were admitted to the wards.

In terms of surgical management, a multi-center study reported a significant delay in diagnostic and therapeutic practices for 70.9% CRC patients. In the delayed patients, 48.9% of respondents reported a change in the initial surgical plan, and 26.3% reported a shift from elective to urgent operations [23]. In addition, postoperative complications occur in half of infected patients, who are associated with increases in mortality rate of up to more than 20% [24, 25]. In our center, through reasonable triaging of surgical procedures, selection of patients, deployment of medical resources, and careful pre-operation and post-operation observations, we maintained a large volume of surgeries as well as surgical safety. Moreover, at the time of writing, there has been no infected patient nor medical staff in our center throughout the pandemic. First, we agree that elective surgeries such as stoma closure can be delayed [16]. Second, palliative surgeries can also be postponed unless the patient experiences serious tumor-related complications that require urgent stoma formation for decompression. Third, owing to the lack of available blood resources, multidisciplinary surgery for CRC with peripheral invasion or liver metastasis is not recommended, as massive bleeding may occur during hepatic surgery. Staged resection combined with chemotherapy is suggested as an alternative. Fourth, curative surgeries should be performed for resectable CRC patients during the pandemic. Close observation and surveillance should be performed both in pre- and post- operation periods, and prolonged hospital stay ensures surgical safety. It is also important to generate a quick workflow to distinguish suggested infected patients from those with postoperative infection and tumor fevers, which has allowed us to identify and treat oncological complications. Last, elderly patients (aged > 70 years) are at a greater risk of infection owing to both their cancer and age [18]. However, we believe that surgery for elderly patients should not be avoided, and regular curative surgery should be performed for those who meet the indications of surgery and infection prevention measures. Finally, preoperative measures taken by our medical staff are in line with the experience of cross regional research [26].

Recently, there is intense debate in surgical societies on the safety of laparoscopy. A few studies reported the detection of SARS-CoV-2 RNA in peritoneal fluid and highlighted the potential risk of aerosol-viral transmission to the medical staff [26, 27]. However, there is also research showing that minimally invasive surgical approaches offer significant advantages with respect to both patient care and the mitigation of viral transmission risk during surgery, provided the appropriate equipment and expertise are present [28]. In our experience, we have no compelling evidence supporting the notion that respiratory or blood-borne infectious viruses can be transmitted through surgical plumes or aerosolized laparoscopic gas. Laparoscopy is less traumatic compared with laparotomy, and the former may expedite recovery when compared with an open procedure. Laparoscopy allows for a self-contained operative field, which reduces the spillage of fluids and tissues, thereby decreasing the risk of operative staff to infection. Thus, we recommend the use of laparoscopy during the pandemic.

We recommend preventive stoma for patients who are at high risk for complications to improve surgical safety in the perioperative period. We also recommend neoadjuvant radiotherapy treatment, in accordance with the European guidelines [29], particularly for elderly patients. Neoadjuvant radiotherapy can contribute to tumor progression control [30] and allow for resource re-allocation, given staffing shortages. Once the pandemic subsides, we will perform curative resection for these patients.

There are some limitations in our research. Firstly, this is a retrospective single-center study. Secondly, the results of our efforts during the pandemic have yet to be confirmed with longer follow-up times.

## Conclusions

In conclusion, during the COVID-19 pandemic, outpatient volumes decreased significantly, especially among the most vulnerable patients including migrants and elderly patients. Nonetheless, we largely maintained the number of patients who received chemotherapy and surgery. Online medical counseling was effective in patient selection and maintaining treatment and follow-up. Adjuvant and palliative chemotherapy for CRC patients should not be discontinued. Elective, palliative, and multidisciplinary surgeries can be postponed, whereas curative surgery should be performed. For elderly CRC patients, endoscopic surgery and neoadjuvant radiotherapy are recommended.

## Data Availability

The datasets used and analyzed during the current study are available from the corresponding author on reasonable request.

## Abbreviations

COVID-19: Coronavirus disease
CRC: Colorectal cancer
CT: Computed tomography
MRI: Magnetic resonance imaging
